# Could well-established, widely available, and simple laboratory techniques explain a laboratory origin of SARS-CoV-2?

**DOI:** 10.1016/j.bjid.2023.102808

**Published:** 2023-10-03

**Authors:** Robert M. Lloyd Jr, James R. Hunter, Ricardo Sobhie Diaz, Mauro Schechter

**Affiliations:** a CorpoRx, Fort Pierce, FL, USA; b Universidade Federal de São Paulo, Escola Paulista de Medicina, Laboratório de Retrovirologia, São Paulo, SP, Brazil; c Universidade Federal do Rio de Janeiro, Departamento de Doenças Infecciosas e Parasitárias, Rio de Janeiro, RJ, Brazil

There has been a renewed interest in the scientific debate on the origin of SARS-CoV-2 (SARS-2). The controversy stems mainly from the alleged need for controversial and sophisticated state-of-the-art gain-of-function technologies. In this commentary, we offer a simple yet plausible explanation by which SARS-2 could have been produced in a laboratory using simple, time-proven, and well-established laboratory technologies.

Lung adenocarcinoma cell line A549, obtained from a 58-year-old Caucasian male, developed in the mid 1970′s, has since then been the standard cell line for in vitro cultivation of coronaviruses, including SARS and MERS.^1^^,^^2^ In 2014, a new Asian human lung adenocarcinoma cell line that could also support the growth of coronaviruses was developed from a 61-year-old female Japanese patient.^3^ Despite similar prognoses, Asian and Caucasian patients with lung adenocarcinomas differ in their cellular biology and mutation patterns in their neoplastic cells.^4^

Serial passages in cell cultures over time select the fittest viruses. The selected viruses are often hypersensitized to the cell line's phenotype in which they were grown. This fundamental principle of cell culture may explain why Asian versus Caucasian/European mortality rates drastically differed in the pandemic's first wave (see below).

Virus genetic recombination is an evolutionary process leading to the emergence of more adapted (fit) virus strains. In vitro genetic recombination is a process by which chimera of two or more virus strains from the same virus family are created through co- cultivation.^5^ The crossover of two nucleic acid strands that share similar sequences can be a mechanism to provide selective bias of new virus strains in human cell lines with distinct receptors. Genetic recombination can occur with both DNA and RNA viruses, including viruses of the *Coronaviridae* families.

It is thus plausible that gain of function could be induced via co-cultivation, serial passage, and in vitro genetic recombination with bat *R. affinis*, RaTG13, and pangolin coronavirus^6^ and thus produce a new lineage of a unique virus with particular receptor requirements. This process would allow for acquired features which, in turn, would allow for selective infection of human cells, depending on the cell lines used for propagation. The resulting virus would become better adapted, and hence less pathogenic, to the new Asian cell line than to the Caucasian cell line traditionally used for the cultivation of Coronaviruses. The presence of furin cleavage sites has fundamental implications for the pathogenicity of SARS, MERS, and SARS-2. It should be noted that fur in cleavage sites are uncommon in bat beta coronaviruses, which might also argue in favor of a laboratory origin of SARS-2.^7^^,^^8^

The simple yet feasible theory we propose on the plausible laboratory origin of SARS-2 is reinforced by the highly statistically significant association between a lower prevalence of specific genetic blood markers in East and South Asian populations and lower mortality in comparison with Western Europeans during comparable periods of the first wave of COVID-19, a time when viral diversity and immunity induced by natural infection and/or vaccination could not have influenced outcomes. A pairwise comparison of these two regions using a Wilcoxon rank-sum test (*W* = 167, p-value < 1.0e-06) demonstrates this and is based on the mean of the mortality rates in Western Europe of 0.258 deaths per 100,000 population compared to an average for the Asian regions of 0.028 per 100,000 population.^9^^,^^10^ It should be noted that, differently from the original strains, the Omicron strains caused high mortality rates in Asian populations but not in Western European populations. The Omicron strains likely originated from SARS-2 strains that jumped from humans to mice (spill back), rapidly accumulated mutations conducive to infecting that host, then jumped back into humans (spill over), indicating an inter-species evolutionary origin.^11^

In summary, it is conceivable that SARS-CoV-2 might have been produced in a laboratory through well-established, widely available, and relatively simple techniques without the need for sophisticated gain-of-function technology. As new evidence continues to emerge, our understanding of the origin of SARS-CoV-2 will continue to evolve.

## Conflicts of interest

The authors declare no conflicts of interest.
